# Practice characteristics and prior authorization costs: secondary analysis of data collected by SALT-Net in 9 central New York primary care practices

**DOI:** 10.1186/1472-6963-14-109

**Published:** 2014-03-06

**Authors:** John W Epling, Emily M Mader, Christopher P Morley

**Affiliations:** 1 Department of Family Medicine, SUNY Upstate Medical University, 750 E. Adams St., MIMC 200, Syracuse, NY 13066, USA; 2 Department of Public Health & Preventive Medicine, SUNY Upstate Medical University, Syracuse, NY, USA; 3 Department of Psychiatry & Behavioral Sciences, SUNY Upstate Medical University, Syracuse, NY, USA

## Abstract

**Background:**

An increase in prior authorization (PA) requirements from health insurance companies is placing administrative and financial burdens on primary care offices across the United States. As time allocation for these cases continues to grow, physicians are concerned with additional workload and inefficiency in the workplace. The objective is to estimate the effects of practice characteristics on time spent per prior authorization request in primary care practices.

**Methods:**

Secondary analysis was performed using data on nine primary care practices in Central New York. Practice characteristics and demographics were collected at the onset of the study. In addition, participants were instructed to complete an "event form" (EF) to document each prior authorization event during a 4–6 week period; prior authorizations included requests for medication as well as other health care services. Stepwise Ordinary Least Squares (OLS) Regression was used to model Time in Minutes of each event as an outcome of various factors.

**Results:**

Prior authorization events (N = 435) took roughly 20 minutes to complete (beta = 20.017, p < .001); Medicaid requests took less time (beta = −6.085, p < .001), and Electronic Health Record (EHR) system use reduced prior authorization time by about 5 minutes (beta = −5.086, p = .002).

**Conclusions:**

While prior authorization events impose substantial costs to primary care offices, it appears that Medicaid requests take less time than private payer requests. Results from the study provide support that Electronic Health Record usage may also reduce time required to complete prior authorization requests.

## Background

In the U.S., health insurers are increasingly requiring that services provided to patients be submitted for prior authorization (PA) [1]. Prior authorization is the requirement to obtain prior approval for the reimbursement of prescription medication, as well as other services including medical procedures, tests and specialist appointments. Prior authorization has been shown in various studies as a potential tool to encourage appropriate, cost-effective utilization of health care services [2]. However, to handle the additional workload of PA tasks, primary care offices must either hire more staff or assign the PA duties to their existing and already overburdened physicians, nurses and clerical staff. Either approach further erodes the tenuous financial margin of primary care practices [3,4]. Although the impact of prior authorization requirements on the provision of specific types of healthcare services has been measured in a variety of specific contexts, there are limited data on the scope and cost of prior authorization for primary care offices. Within a small body of literature on physicians’ non-clinical practice costs, prior authorization has been studied only as a subcategory with limited data. These studies are either very broad and do not distinguish prior authorization tasks, focus on very specific types of events, such as one payor/one drug [5], or do not include the often substantial time cost to the practice for the prior authorization work of non-physician staff [6-9]. A single study that focused on prior authorizations [10] was not performed in a primary care context, and was focused on prior authorization for medications only. Three more recent studies have examined physician practice interactions with health insurers and have included prior authorization in their analysis. In a national survey conducted in 2008 by Casalino and colleagues, physicians reported spending three hours weekly interacting with insurance plans, with additional time spent by nurses, medical assistants, and clerical staff [11]. When time was converted to dollars, Casalino estimated that the national cost to practices of interactions with health plans is at least $23 billion to $31 billion each year. Similarly, Sakowski and colleagues conducted a national study on the cost of support personnel to address billing and insurance issues in U.S. primary care practices [12]. They estimated that $85,276 was spent on personnel costs per full time equivalent (FTE) physician, or approxmiately10 percent of practice revenue. In 2011 Morra and colleagues estimated a similar amount, $82,975 per U.S. physician FTE, or nearly four times the amount spent per physician FTE in Canada [13]. These studies support the belief that the prior authorization burden is substantial and that it is, therefore, an important target for health reform. Both studies, however, lack the granularity to suggest specific solutions for reducing the PA workload and determining cost. Additionally, each relied on self-reporting of recalled events. Nevertheless, the American Medical Association has called for standardization of prior authorization costs in response to the administrative and financial burdens that appear to be growing annually [14].

Aside from the studies by Casalino, Sakowski and Morra, physicians in practice have become distressed by what they perceive to be an additional workload and inefficiency imposed by the increasing PA requirements. During a free-form brainstorming session at the 2008 convocation of the Studying-Acting-Learning-Teaching Network (SALT-Net), physicians in the audience were asked to respond to a prompt about what was “bugging them in practice.” Without any prompting, the effects of PA were readily apparent. In response to this local impetus, we designed and carried out a prospective, concurrent, self-report study of primary care prior authorization activities to document the costs that a sample of primary care practices incurred performing required prior authorization activities. We also sought to identify any practice characteristics that may influence the time allocation and costs of prior authorization activities for these practices, such as staff composition and the use of electronic health record (EHR) systems.

The initial results of that study, conducted in 9 practices in the central part of New York State, indicated that the mean projected annual cost per full-time equivalent (FTE) physician was $2,161.75 (range, $926.13–$4,604.11) [15]. Given the distance between those results, and the previous recall-based reports, we have proceeded with a secondary round of inferential analyses of the data collected, and report these secondary results here.

## Methods

Practices were recruited from SALT-Net by an introductory letter. Once enrolled, practices were asked to fill out a practice survey that asked about practice demographics, payer mix, staffing ratios and office technology. Recruited practices were trained (on-site in six cases, and via teleconference for three distant practices) in the use of a data collection card (the “event form” or EF) and other study procedures. The EFs were used to document data on the medical service requiring prior authorization; the category of insurance provider authorizing the intervention; the category of staff member documenting the activity (provider, nurse, office assistant, clerical, etc.) and the time spent on the activity. The services requiring prior authorization within these practices included medication, laboratory testing, radiologic testing, medical equipment, nursing, occupation and physical therapy, and other procedures; data on specific medications requested were not collected in this study. Participants (clerical staff, nurses and physicians/nurse practitioners/physician assistants) were instructed to document consecutive prior authorization requests (1 per event form) within one month and were asked to note the stop and start dates of their recording period. Each study participant completed an EF for every prior authorization request they worked on during a 4–6 week period in the fall of 2010. The study was stopped after 6 weeks at each site, regardless of number of EFs completed per staff member; some of the practices experienced data collection periods shorter than six weeks. Where single PA requests required activity by multiple participants, each participant recorded their time individually on a separate EF. Thus, it is important to note that the actual number of PA requests submitted by each practice was not collected; rather, the EF forms were used to document the number of staff working on PA requests; the time staff devoted to PA request activities; and general characteristics about the staff working on PA requests and PA activities. At completion of the study, we asked the practices to estimate the percentage of prior authorization requests they actually captured in the EFs during the study. This study was reviewed and approved by the Institutional Review Board for the Protection of Human Subjects at SUNY Upstate Medical University.

Data from the EFs were entered into a Microsoft Excel (r) (version 2003) spreadsheet, and a 10% random data entry validity check was performed. Data were analyzed primarily by hours per week spent on prior authorization activities. This was stratified by staff type, insurance type and type of service requested. Average PA activity cost per practice per week (based upon US average) was calculated using salary data from the US Bureau of Labor Statistics (USBLS) for 2010 as well as the time data obtained from EFs. Costs per request were estimated by multiplying the USBLS 2010 average hourly wage for the staff type involved in the request by the time recorded on the EF as spent on the request by the staff member. The total was then multiplied by a factor of 1.25, to account for fringe benefits and other compensation costs (based upon a personal communication regarding estimates for fringe costs of primary care physicians, mid-level providers, and medical office staff with Nick A. Fabrizio, PhD, MGMA Medical Management Consultant).

To assess the effects of interactions (i.e. type of insurer, purpose of request, respondent and practice characteristics) on the time required for each prior authorization, time spent on PA requests was calculated in minutes and modeled as a dependent variable, where each EF was treated as a case. Stepwise Ordinary Least Squares (OLS) Regression was used to model Time in Minutes of each event as an outcome of the following factors: practice; reporter type (Physician, NP, PA, RN, LPN, Clerical); insurance type involved in the transaction (Medicare, Medicaid, Commercial, Worker Compensation, Pharmacy Benefits); purpose of PA request (Medication, Procedures, Radiology, OT/PT, Nursing Care, Medical Equipment); rurality of practice (Rural Urban Commuting Area [RUCA] code >7 = 1 [16]; and full implementation of an Electronic Health Record (EHR). The stepwise construction of the regression model resulted in the inclusion of only those variables that were statistically meaningful in the prediction of time required to complete each PA request. Reference cases were the home practice of the investigators (the practice that provided the most responses), physician as the reporter, and “not recorded” for insurance and purpose. The use of practice and reporter type as variables was selected to control for hierarchically nested effects in a less computationally demanding and more easily interpretable format than using more intensive methods, such as general estimating equations or mixed model procedures. A Durbin-Watson statistic was calculated for the model to test for autocorrelation between the cases, as the matrix was stacked by practice and within-practice respondent. Additionally, with the number of independent variables included in the model, multicollinearity diagnostic statistics were computed.

## Results

Nine practices registered for and completed the study. Within those practices, sixteen clinicians (11 physicians, 4 physicians’ assistants and 1 nurse practitioner), 17 nurses (7 registered nurses and 10 licensed practical nurses) and 13 clerical staff contributed at least one EF to the study. Table 1 displays the practice characteristics for each of the nine practices included in this study. Each of these characteristics was incorporated in the stepwise regression model as independent covariates. Six of nine practices estimated that they captured > 90% of their prior authorization data. The other three practices estimated 50-70% capture, largely because of individual reporters within the practice who failed to report their data consistently. It is important to note that these were informal estimates and may not reflect the true capture of PA activities for each practice. Furthermore, since some practices collected data in periods of less than six weeks, the amount of EFs collected may not reflect the true amount of PA activities conducted across the full six week period of this study.

**Table 1:** Practice characteristics

							**% Patients in age group**			
**Practice**	**No. patients**	RUCA code*	**Ownership**	**% Medicaid**	**EHR?†**	**% Male patients**	**14 and under**	**15-44**	**45-64**	**65+**
*1*	10000	1	Physician/physician group	28	Yes	40	----	----	----	----
*2*	13200	7	Physician/physician group	18	No	40	27	27	22	24
*3*	10000	2	Physician/physician group	0.2	Yes	45	10	20	40	30
*4*	7000	3	Physician/physician group	6	Yes	40	22	35	25	18
*5*	3155	1	Physician/physician group	11	Yes	41	7	38	32	23
*6*	5000	10.4	Physician/physician group	12	Yes	50	35	20	15	30
*7*	3000	10.4	Hospital	7	No	40	20	20	20	40
*8*	7136	2	Physician/physician group	27	Yes	40	17	45	27	11
*9*	1460	7	Physician/physician group	1	Yes	40	11	42	36	11

*RUCA = Rural Urban Commuting Area, †EHR = Electronic Health Record.

There was a wide variation in Medicaid insurance percentage among practices. The nine practices submitted 442 cards. Data for 435 cards were analyzed (7 cards had insufficient data recorded). Table 2 presents each type of service as a percentage of all services requested in PA activities, as reported on the EFs per practice. The mean projected annual costs per FTE, based upon USBLS 2010 average salaries adjusted for a 25% fringe benefit rate, was $2,161.75 (range: $926.13 to $4,604.11) for the nine practices. We stratified the average time and cost associated with PA requests per practice per week by staff category (Table 3), service requested (Table 4) and insurer (Table 5). Clerical staff and RNs spent the largest amount of time working on PA requests, and PA requests for medication and radiologic testing accounted for the largest amount of time devoted to PA activities out of all services requested. The largest costs associated with PA requests were due to clerical and physician time spent on prior authorization activities, activities concerning medications and radiology procedures and because of commercial insurance and interactions with staff members at pharmaceutical insurance plans (i.e. pharmacy benefit managers). While physicians did not spend the most amount of time working on PA requests, their time spent working on PA activities accounted for a larger proportion of costs due to increased salary rates.

**Table 2:** Type of service as a percentage of all PA services requested (EF per practice)

**Practice**	**Medical equipment**	**Medication**	**Nursing**	**Procedure**	**OT/PT**	**Radiology**	**Not recorded**
*1*	0	16.5	0	4.9	44.7	34.0	0
*2*	0	44.9	0	2.0	4.1	44.9	4.1
*3*	1.6	25.8	0	0	6.5	66.1	1.6
*4*	2.0	52.0	0	16.0	6.0	26.0	0
*5*	0	76.3	0	0	0	23.7	0
*6*	3.6	71.4	0	0	0	28.6	0
*7*	0	19.0	57.1	0	0	23.8	0
*8*	0	50.0	0	3.1	0	46.9	0
*9*	0	12.2	0	0.	0	87.8	0

**Table 3:** Cost of prior authorization requests over a four-week period

**Practice**	**Total EF count**	**Total time in minutes**	**Mean time per request (minutes)**	**Projected annual cost ($)**	**Mean # request/week**	**Per FTE Physician**				
						**FTE**	**Requests/week**	**Time/week**	**Cost/week**	**Cost/year**
*1*	103	972	9.4	3804	25.8	4.5	5.8	54.6	18	$926
*2*	49	874	17.8	5020	12.3	3.0	4.1	72.8	35	$1,813
*3*	63	899	14.3	5112	15.8	5.0	3.2	45.0	21	$1,108
*4*	51	1,231	24.1	4647	12.8	3.0	4.3	102.6	32	$1,678
*5*	38	582	15.3	4250	9.5	1.0	9.5	145.5	89	$4,604
*6*	29	375	12.9	1979	7.3	3.0	2.4	31.3	14	$715
*7*	21	382	18.2	1968	5.3	2.0	2.6	47.8	21	$1,066
*8*	32	434	13.6	2540	8.0	1.5	5.3	72.3	35	$1,835
*9*	49	735	15.0	5278	12.3	3.0	4.1	61.3	37	$1,906

**Table 4:** Cost by prior authorization

**Pre-cer**	**EF count**	**Time (minutes)**	**Average time (minutes)**	**Estimated cost ($)**	**Cost (%)**
*Medical equip*	3	55	18.3	62	1.7
*Medication*	156	2,433	15.6	1648	45.7
*Not recorded*	3	40	13.3	19	0.5
*Nursing*	12	170	14.2	69	1.9
*OT/PT*	55	374	6.8	137	3.8
*Procedure*	15	292	19.5	113	3.2
*Radiology*	191	3,120	16.3	1555	43.2

**Table 5:** Cost of prior authorization requests by staff role

**Position**	**EF Count**	**Total time (minutes)**	**Time (%)**	**Average time per EF (minutes)**	**Cost (%)**	**Cost estimate ($)**	**Average cost ($)**
*Clerical*	221	3147	48.5	14.2	18.7	1053	4
*LPN*	79	983	15.2	12.4	16.3	400	4
*PA*	20	298	4.6	14.9	19.6	270	11
*Physician*	34	532	8.2	15.6	20.6	852	20
*RN*	81	1524	23.5	18.8	24.8	1029	10

Results of the regression analysis indicate an expected value of slightly more than 20 minutes spent (β = 20.017, p < .001) for each PA request. Medicaid requests took approximately 14 minutes (β = −6.085, p < .001), and full implementation of an EHR reduced the average time for PA activities by slightly more than 5 minutes (β = −5.086, p = .002). One practice had longer than expected values for PA request times (β = 11.389, <.001), although this practice had several outlier cases, including one that took roughly 115 minutes to accomplish. Requests for prior authorization of occupational or physical therapy were processed much more quickly than the general case, by almost 9 minutes (β = −8.716, p < .001). Almost all cases involving OT/PT requests were made by one practice. Regression results are presented in Table 6.

**Table 6:** Output of stepwise OLS regression, modeling the effects of practice, EHR* usage, purpose of prior authorization request, insurance type involved, reporter type, and rurality of the practice location

**Variable**	**Beta**	**t**	**p**	**Lower bound (95% CI)**	**Upper bound (95% CI)**
*Constant*	20.017	13.018	<.001	16.995	23.040
*Practice X*	11.389	6.108	<.001	7.724	15.054
*Purpose - OT/PT†*	−8.716	−4.847	<.001	−12.250	−5.182
*Medicaid*	−6.085	−3.871	<.001	−9.174	−2.995
*EHR in use*	−5.086	−3.085	.002	−8.327	−1.845

Model Parameters: R^2^ = .154; F = 19.506, p < .001.

Dependent Variable: Time (in minutes) of each Prior Authorization event.

Independent Variables removed from equation: All practice indicator variables aside from one (Practice X); all purposes for PA calls aside from OT/PT; all other insurance types aside from Medicaid; rurality; all reporter types (physician, nurse, clerical, etc.).

*EHR = Electronic Health Record, †OT = Occupational Therapy, PT = Physical Therapy.

## Discussion

Prior authorization requirements have real, measurable costs, as previously described [15]. However, in our small, geographically limited study, there appear to be factors that mitigate these costs. PA requests for Medicaid appear to take less time to complete, which may be indicative of either a more efficient process relative to other payers, or possibly more scrutiny or complexity on the part of private payers. Additionally, the use of electronic health record systems appears to influence the amount of time, and hence costs, for each PA request activity. A comparison of mean time needed to complete a PA request among the different staff within each practice revealed that RNs and clerical staff utilizing EHRs took less time to complete PA requests than RNs (p = .027) and clerical staff (p < .001) not using EHRs (although clerical staff using EHRs vastly outnumbered those not using an EHR); there was no statistical difference in completion times among other staff types. This finding may represent either a benefit of increased administrative efficiency from EHR implementation, or a more general characteristic common to practices that had implemented EHR systems by 2010.

In 2009 Casalino et al. estimated through a self report survey that primary care physicians spent an average of 1.1 hours per week on prior authorization activities, clinical staff (RN/LPN/MA) spent 19.8 hours/week/FTE physician, and clerical staff spent 35.9 hours/week/FTE physician. However, our study found a much lower time requirement, with physicians spending much less than an hour/week/FTE physician (0.2 hour/week). Other clinical staff (PA/RN/LPN/MA) spent as little as 0.45 hour/week on average, while clerical staff seemed to spend the most amount of time (0.5 hours/week/FTE physician). It is important to note that while the number of FTE physicians per practice is highly correlated with the overall number of completed EFs, this variable is not correlated with the mean times observed for each of the staff types (data not shown). It appears the estimates for amount of time spent by each staff member varies by staff member, by office procedures, and/or other factors more than simply by the number of FTE physicians. Additionally, the current study found vastly different estimates of projected annual costs per FTE physician than the studies by Morra [13] and Sakowski [12].

There were several findings worthy of additional study in larger, more diverse samples. First, EHR usage appears to reduce time devoted to prior authorization requests. The current results provide additional evidence for the benefit of electronic health records [17,18]. A larger sample may be useful in determining a refined estimate of effect size. Additionally, it is worth verifying whether Medicaid prior authorization procedures are truly more efficient, from the perspective of time commitment by practices, and if so, whether such efficiency gains are made without sacrificing legitimate review for cost-control and appropriateness of request.

There are several weaknesses inherent in this study. First, the participating practices comprised a convenience sample, consisting of those willing to participate in the study. Given the noted enthusiasm for further study of the topic, those who chose to participate in this study may over-represent adversely affected practices and may not reflect the experiences of those practices that were not part of the study. Additionally, the study was geographically limited. The estimates reported above are, therefore, not nationally generalizable estimates. However, we are confident that the practices participating in this study are qualitatively representative of primary care practices in the central New York region in terms of experience, size, and organization. Furthermore, it is also probable that not all prior authorization events were recorded, as some practices may have viewed the completion of the EFs as burdensome. Thus, the estimates of >90% participation as well as the number of PA requests completed by clinical staff may be inaccurate, particularly among physicians who experience increased time constraints. A larger sample would have allowed more sophisticated analytic techniques and greater generalizability. However, we believe that the use of variables to control for hierarchical effects, combined with adequate testing for autocorrelation and multicollinearity, represent the best analysis possible with a small sample size. Furthermore, we believe that the study design represents a step forward, in terms of validity owing to its use of real-time data collection, as opposed to recall surveys.

Finally, as we have noted previously, the costs and overall time commitments reported above are direct costs and do not take into account lost opportunity costs [15]. For example, the commitment of a clerical staff member for 2–3 hours per week to prior authorization requests may not represent a large financial outlay or a large percentage of the staff person’s work time. However, the same staff person may be pulled away from other more appropriate duties and may lose time starting and stopping prior authorization request activity that is not accounted for by the time recorded by the participant. When a potential revenue generator (such as a physician or mid-level provider) is drawn into prior authorization activities, the opportunity and efficiency costs may be much greater. Also, delays in treatment or diagnoses were not captured or calculated as a consequence of prior authorization barriers in either study, but such harm most certainly occurred and continues today. The present study does not capture or attempt to estimate such costs, and additional data collection and analysis is warranted. We suspect that our study under-estimates the true impact of prior authorization requirements on practices, clinician, and patients. The actual dollar losses reported, if one includes opportunity costs, are most likely underestimates. The results presented here should be interpreted with downstream costs in mind. In relation to the results found by Casalino [11], Sakowski [12], and Morra [13], where annual costs exceed $80,000 per FTE, the low cost findings presented here and by our group previously [15] should be viewed as a lower bookend, with true costs lying somewhere within the range of results found by all four studies.

## Conclusions

Where previous studies examining prior authorization have experienced issues surrounding recall bias, this study presents data that more closely approximates the negative impact of prior authorization on primary care offices through the use of real time data collection via direct self-observation. Thus, these findings complement the estimates from previous studies by providing the granularity that is needed to inform both the practice and legislative communities about the burden of prior authorization activities on primary care practices [19]. It is also important to consider that prior authorization requirements may yield benefits, such as cost savings to the system [20] or the promotion of rational prescribing trends [21], although to date, the literature on this point appears to be equivocal [2,22-27]. In light of the pivotal role of primary care now and in the future [28-30], the benefits and costs of prior authorization activities in primary care deserves further understanding and action, and primary care offices should be appropriately compensated for this work. While the previously stated limitations narrow the implications of this study, these findings should be viewed as a starting point in the development of future investigations.

## Competing interests

The authors declare that they have no competing interest.

## Authors’ contributions

JWE and CPM designed study protocol and oversaw practice recruitment and data collection. CPM wrote the first draft of the manuscript, and conducted the initial analysis of the data. EMM conducted additional analyses. JWE, CPM, and EMM interpreted the analyses and edited the final document. All authors read and approved the final manuscript.

## Pre-publication history

The pre-publication history for this paper can be accessed here:

http://www.biomedcentral.com/1472-6963/14/109/prepub
